# Positive Effects of bFGF Modified Rat Amniotic Epithelial Cells Transplantation on Transected Rat Optic Nerve

**DOI:** 10.1371/journal.pone.0119119

**Published:** 2015-03-03

**Authors:** Jia-Xin Xie, Yu Feng, Jian-Min Yuan, Zhen-Dong You, Hai-Yan Lin, Chang-Lin Lu, Jia-Jun Xu

**Affiliations:** 1 Department of Anatomy, The Second Military Medical University, Shanghai, P. R. China; 2 Department of Neurobiology, The Second Military Medical University, Shanghai, P. R. China; 3 Department of Orthopaedics, Changzheng Hospital, The Second Military Medical University, Shanghai, P. R. China; 4 People's Liberation Army Clinical Center for Spinal Cord Injury, Kunming General Hospital of Chengdu Military Command, Kunming, P. R. China; Harvard University, UNITED STATES

## Abstract

**Purpose:**

Effective therapy for visual loss caused by optic nerve injury or diseases has not been achieved even though the optic nerve has the regeneration potential after injury. This study was designed to modify amniotic epithelial cells (AECs) with basic fibroblast growth factor (bFGF) gene, preliminarily investigating its effect on transected optic nerve.

**Methods:**

A human bFGF gene segment was delivered into rat AECs (AECs/hbFGF) by lentiviral vector, and the gene expression was examined by RT-PCR and ELISA. The AECs/hbFGF and untransfected rat AECs were transplanted into the transected site of the rat optic nerve. At 28 days post transplantation, the survival and migration of the transplanted cells was observed by tracking labeled cells; meanwhile retinal ganglion cells (RGCs) were observed and counted by employing biotin dextran amine (BDA) and Nissl staining. Furthermore, the expression of growth associated protein 43 (GAP-43) within the injury site was examined with immunohistochemical staining.

**Results:**

The AECs/hbFGF was proven to express bFGF gene and secrete bFGF peptide. Both AECs/hbFGF and AECs could survive and migrate after transplantation. RGCs counting implicated that RGCs numbers of the cell transplantation groups were significantly higher than that of the control group, and the AECs/hbFGF group was significantly higher than that of the AECs group. Moreover GAP-43 integral optical density value in the control group was significantly lower than that of the cell transplantation groups, and the value in the AECs/hbFGF group was significantly higher than that of the AECs group.

**Conclusions:**

AECs modified with bFGF could reduce RGCs loss and promote expression of GAP-43 in the rat optic nerve transected model, facilitating the process of neural restoration following injury.

## Introduction

The optic nerve derives from the embryonic optic stalks in the optic vesicles located in the diencephalon; therefore, the microenvironment within the optic nerve is the same as within the central nervous system (CNS), which contains astrocytes, oligodendrocytes, microglias. Axons of the RGCs form optic nerve, however, once injury happens the axons will ordinarily fail to regrow through the lesion site while the RGCs will die gradually, leading to permanent vision loss [1]. The failure of the optic nerve to regenerate after injury or in neurodegenerative diseases remains a major clinical and scientific problem. However, the injured RGCs axons were proven to be able to regenerate in certain extent, which made it possible for improving the regenerative potential of the optic nerve [2, 3].

Basic fibroblast growth factor (bFGF) which is a member of the fibroblast growth factor family plays a critical role in the development of the retina, and has been identified as a potent stimulator of axon growth for developing and regenerating RGCs [4]. Several animal studies have indicated that the application of bFGF could possiblely promote the survival of RGCs, and stimulates regrowth of the axons within the injured optic nerve [5, 6].

Amniotic epithelial cells (AECs) are differentiated from amnioblasts 8 days after fertilization [7]. It was proven that AECs not only could secrete several neurotrophic factors, such as brain-derived neurotrophic factor (BDNF) and neurotrophin-3 (NT-3), but also retain stem cell characteristics [8,9]. Moreover AECs had been applicated as donor cells of cell therapy in brain and spinal cord diseases, revealing that AECs have certain influence on promoting neural regeneration [10,11]. Accordingly, the combination of AECs with bFGF may theoretically be beneficial in ameliorating the microenvironment of the injured optic nerve, enhancing neural regeneration.

In the present study we established rat AECs modified with human bFGF gene utilizing lentivirus, and confirmed expression and secretion of bFGF in the genetically engineered AECs culture. Whereafter we transplanted the genetically engineered AECs into the transected optic nerve in rat, observing the effects of the cells on RGCs and the injured optic nerve.

## Materials and Methods

### Experimental Animals

Adult Sprague-Dawley rats were used in this study. The animals were housed in seperated cages in a temperature-controlled room with a 12 h light/dark cycle and free access to food and distilled water. All the protocols were approved by the Administrative Committee of Experimental Animal Care and Use of Second Military Medical University, China (SMMU, Licence No. 2011023). The animals had received humane care throughout all the procedures in accordance with the Guide for the Care and Use of Laboratory Animals, published by the US National Institutes of Health (Publication No. 85-23, revised 1996). In addition the ARRIVE (Animals in Research: Reporting In Vivo Experiments) guidelines was followed as well.

### Rat AECs Cultures

The rat amniotic membrane was collected from deeply anesthetized pregnant rats at 19 days of pregnancy. The collected membrane was then treated with 0.25% trypsin (Gibco) 3 times, each for 20 min. AECs were collected and cultured in Dulbecco's modified Eagle medium (DMEM, Gibco) supplemented with 10% fetal bovine serum at 37°C with 5% CO_2_.

### Construction of Rat AECs Modified with Human bFGF Gene

A bFGF gene segment (556bp) was synthesized according to human bFGF (hbFGF) cDNA sequences (GenBank, NM_002006.4). The restriction enzyme sites: Spe I and Xho I were introduced into the segment. Afterwards, plasmid vector pLenti6/V5-GW/lacZ (Invitrogen) was digested with restriction endonuclease Spe I and Xho I to remove lacZ segment, then linked with bFGF gene segment, constructing plasmid vector pLenti6/V5-GW/hbFGF, which contains blasticidin resistance gene within the sequences.

Packaging of recombinant lentivirus was performed utilizing the ViraPower Lentiviral Expression Systems (Invitrogen), and viral supernatant was harvested, storing at −80°C. The titer of the viral supernatant were determined by infecting the human fibrosarcoma cell line HT1080 (Invitrogen) and selected with blasticidin (Invitrogen).

The fourth generation passage rat AECs were seeded into 6-well plates at 1×10^6^/ml to grow as 85% confluence. Afterwards the medium was replaced by mixed medium containing 500 μl viral supernatant and 10 μg/ml polybrene (Sigma), and one control well that containing normal medium in each plate was set. After incubating for 6h, the infectious medium was removed, and the cells were cultivated with fresh medium for 24h. Then the cells were selected in blasticidin (10 μg/ml) selection medium about 12d till all of the cells in control wells died out, and the survived cells were further cultured.

### Gene Expression of bFGF in Transfected AECs

After blasticidin selection, total RNA was extracted from AECs/hbFGF of the third passage. Expression of human bFGF mRNA was detected by RT-PCR. The sequences of bFGF primers used were (5’→3’): forward GAGCGACCCTCACATCAA, reverse CGTTTCAGTGCCACATACC. The products (222bp) were determined by agarose gel electrophoresis.

Meanwhile, culture medium of the first-passage AECs/hbFGF was collected separately 3, 6, 9 and 12 days after culturing, and was tested for bFGF concentration by ELISA.

### Cell Preparation before Transplantation

10d after blasticidin selection, AECs/hbFGF and the same period subcultured AECs that were not infected by lentivirus were chosen for transplantation. On the day of transplantation, both AECs and AECs/hbFGF that grew as 95% or more confluence were trypsinized and made into single cell suspension. The cell density was 8×10^6^/ml, determined by cell counting.

For the purpose of tracing transplanted cells, a certain number of animals (n = 3) in each cell transplantation groups were chosen to receive transplantation of AECs/hbFGF or AECs stained with Hoechst dyes (Hoechst33342, Sigma). 24h before transplantation, the cells were cultured in fresh medium with Hoechst (0.5 μg/ml) for 1h, and then were cultured with common medium till being prepared for transplantation as described previously.

### Grafting Surgery

In the optic nerve transected model, the rat was anesthetized by intraperitoneal injection of 2% pentobarbital sodium (40mg/kg). The optic nerve was exposed through supraorbital approach under microscope, and a clear transverse gap (about 0.5mm) was made 2mm retrobulbarly by sucking the nerve fibers with a micro glass pipette through a small vertical breach on the meningeal sheath, without damaging the vessels [12]. The incision was sutured and the rat was allowed to recover under observation. Ketamine had been applied by intraperitoneal injection (10mg/kg) within 72h post surgery to relieve pain once a painful behavior, such as groaning, refusing to eat or drink, and hypopraxia, was observed. None of the operative complications had taken place during the experiment.

Adult male Sprague-Dawley rats weighing 150–180 g were randomized into the following 4 groups and then allocated to the following protocols:

1. Normal group: sham surgery, the optic nerve was exposed without transection (n = 18)
2. Control group: 10μl DMEM/F12 medium was injected into the transverse gap of the optic nerve (n = 21)
3. AECs group: 10μl AECs suspension was injected into the transverse gap of the optic nerve (n = 21)
4. AECs/hbFGF group: 10μl AECs/hbFGF suspension was injected into the transverse gap of the optic nerve (n = 21)

Within the AECs group and AECs/hbFGF group, 3 rats were transplanted with cells stained with Hoechst dyes separately.

### Tissue Processing and Morphological Evaluations

#### 1. Frozen section of optic nerve

28d after transplantation, the rats in the AECs group, AECs/hbFGF group which were transplanted with cells stained with Hoechst (n = 3) and 3 rats in the control group were sacrificed and fixed by cardiac perfusion with 4% paraformaldehyde. Subsequently, the injured optic nerves were taken out for a 6h post-fixation with 4% paraformaldehyde followed by dehydration with 20% sucrose solution. Afterwards, the optic nerve specimens were made into frozen sections (10 μm) for observation by fluorescent microscope.

#### 2. Fluorescence labeling, Nissl staining and counting of RGCs

Some of the rats (n = 6) in each group were chosen to receive RGCs labeling with BDA, which were conjugated with fluorescein (Vector). 26d after surgery, the rats were anesthetized by peritoneal injection of 2% pentobarbital sodium (40mg/kg), and 5μl 10% BDA was injected into vitreous body with a microinjector. 48h later, the rats were sacrificed and fixed by cardiac perfusion with 4% paraformaldehyde, and then the eyeballs were taken for retina flattening to count BDA labeled cells. Each retina was detached from the eyeball, and was cut with scissors into upper, lower, nasal and temporal pieces according to the conjunctival ink mark. Afterwards, the directionally cut retina was flattened on the slide for RGCs counting under fluorescence microscope. High power fields (40×10) were chosen and were photographed at random on upper, lower, nasal and temporal pieces. For each area, 3 sites which were 1/6, 3/6 and 5/6 distance from optic nerve head were chosen, and 2 high power fields were photographed at random. Only cells with an intact membrane profile and plump cytoplasm, as well as bright fluorescence within cytoplasm were included. The number of labeled cells present in the high power fields was counted and analyzed.

Meanwhile a same number of rats (n = 6) in each group were sacrificed and the retinas were collected for Nissl staining with toluidine blue. The number of RGCs in which the Nissl’s bodies were stained blue were counted and analyzed as the same method described previously, and only cells with an intact membrane profile and plump cytoplasm, as well as hyperchromatic Nissl’s bodies were included.

#### 3. Immunohistochemical staining of GAP-43

28d after surgery, the rats (n = 6) were sacrificed and fixed, and the injured optic nerves were collected and embeded in paraffin. Afterwards, serial sections (5μm) were made for immunohistochemical staining. The thin paraffin sections were proceeded to react with primary GAP-43 antibody (1:50) (CALBIOCHEM) and further react with secondary biotinylated IgG (1:200) (CALBIOCHEM). The immunoreactivity was visualized with streptavidin biotin-peroxidase complex method. Then the sections were observed under microscope, and the injured sites in each group were photographed (20×10) to measure the integral optical density value of the immunoreactive substance with Biosens Digital Imaging System (Shanghai Bio-tec).

### Statistical analysis

All results were expressed as means ± S.E.M. Differences between groups were determined with one-way ANOVA by SAS software (8.2). A value of *P*<0.05 was considered to denote statistical significance.

## Results

### Establishment of bFGF-expressing Rat AECs with Lentiviral Vectors

#### 1. Construction of pLenti6/V5-GW/hbFGF plasmid vector

**Fig 1:**
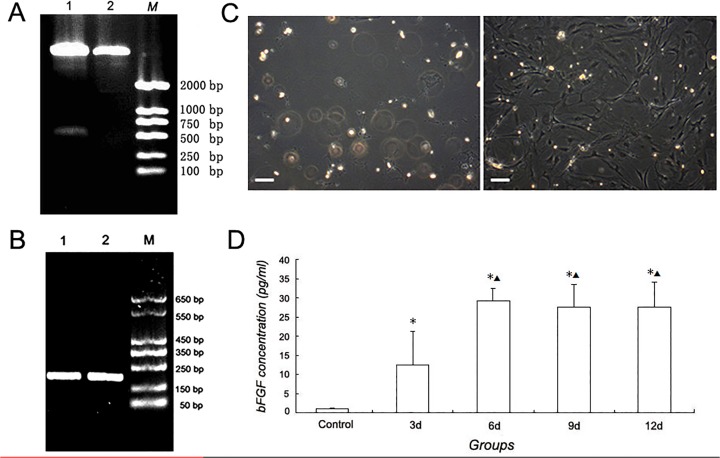
Establishment and confirmation of AECs/hbFGF with lentiviral vectors. A: Enzyme digestion analysis of lentiviral vector. Lane 1: SpeⅠand XhoⅠdouble digestion of lentiviral vector; Lane 2: Undigested lentiviral vector; M, DL2000 marker. B: Expression of bFGF in transfected AECs by RT-PCR. Lane 1 and 2: bFGF segment(222 bp); M: DL2000 marker. C: Morphology of AECs after blasticidin selection. Left: AECs in the untransfected group died and detached from the bottom; Right: Most AECs transfected with target fragment survived. Bar = 20μm D: Concentration of bFGF secreted by transfected AECs in different time groups by ELISA. (*: *P*<0.01 vs Control. ▲: *P*<0.01 vs 3d).

The hbFGF gene segment within plasmid vector pLenti6/V5-GW/hbFGF was confirmed by restricting enzyme digestion and DNA sequencing. A bright electrophoresis strip appeared at approximately 550bp (Fig. 1A). Furthermore, The DNA sequencing analysis proved that the DNA sequence of the segment was in accordance with the hbFGF listed in GenBank (NM_002006.4).

#### 2. Lentiviral infection of rat AECs

Viral supernatant harvested with the human 293FT cells was utilized to infect the HT1080 cells for detection of the viral titer. The titer of the packaged recombinant lentivirus was determined as 3.2×10^5^ TU/ml. The fourth generation passage rat AECs infected with the tittered viral supernatant were cultured in blasticidin selection medium till the uninfected AECs of the control well died out (Fig. 1C).

#### 3. bFGF gene expression and peptide secretion in transfected AECs

After selection with blasticidin, transfected rat AECs were further cultured, in which bFGF was proved to be expressed by RT-PCR (Fig. 1B). Furthermore, concentration of bFGF secreted by transfected AECs at 3, 6, 9 and 12 days after culturing were determined by ELISA (Fig. 1D). The overall difference among groups was statistically significant proven by one-way ANOVA (F = 19.24,*P*<0.0001). Comparing the differences of bFGF concentration in different time groups and the control group by Dunnett-t Test, it was indicated that bFGF concentrations in different time groups were higher than the control group (3d group *VS* control: *P* = 0.030, others: *P*<0.0001), and the bFGF concentration in 6, 9, 12d groups were higher than that of the 3 d group (6d *VS* 3d group: *P* = 0.001, 9d *VS* 3d group: *P* = 0.003, 12d *VS* 3d group: *P* = 0.003) (Fig. 1D).

### Rat Model of Optic Nerve Injury

**Fig 2:**
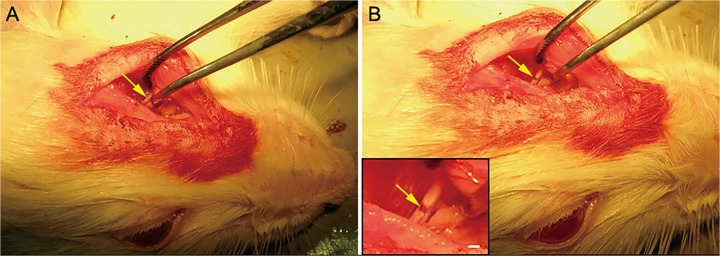
Rat model of optic nerve transection. A: Exposed rat optic nerve (arrows). B: The transverse gap in the transected optic nerve (arrows). Bar = 500μm.

The optic nerve of rat was exposed surgically and sucked with a micro glass pipette, leaving a 0.5mm clear transverse gap (Fig. 2). Since the meningeal sheath was reserved well and vessels were kept intact, it was possible to inject cell suspension into the transverse gap and keep it. This rat model provides an ideal tool to investigate nerve regeneration following complete optic nerve injury [12].

### Transplanted AECs Survived and Migrated within the Injured Optic Nerve

**Fig 3:**
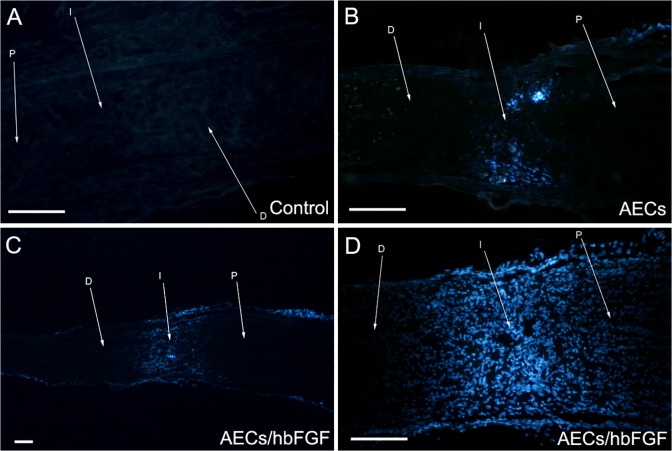
Tracing transplanted cells with Hoechst dyes within the injured optic nerve 28d after transplantation. A: The transected optic nerve without cell transplantation. B: The transected optic nerve with AECs transplantation. AECs survived within the injury site. C: The transected optic nerve with AECs/hbFGF transplantation. AECs/hbFGF survived and migrated within the injury site (40×). D: The transected optic nerve with AECs/hbFGF transplantation. AECs/hbFGF survived and migrated within the injury site (100×). Bar = 300μm. (I: Injured area; P: Proximal part; D: Distal part).

Observation of optic nerve frozen section showed that 28d after transplantation, both AECs/hbFGF and AECs that stained with Hoechst were still alive within the lesion site. However, the AECs/hbFGF survived more robustly and migrated along with the residual glial cells to a distance of 2–3mm from the transected site (Fig. 3).

### AECs Transplantation Reduced Post-traumatic RGCs Loss

**Fig 4:**
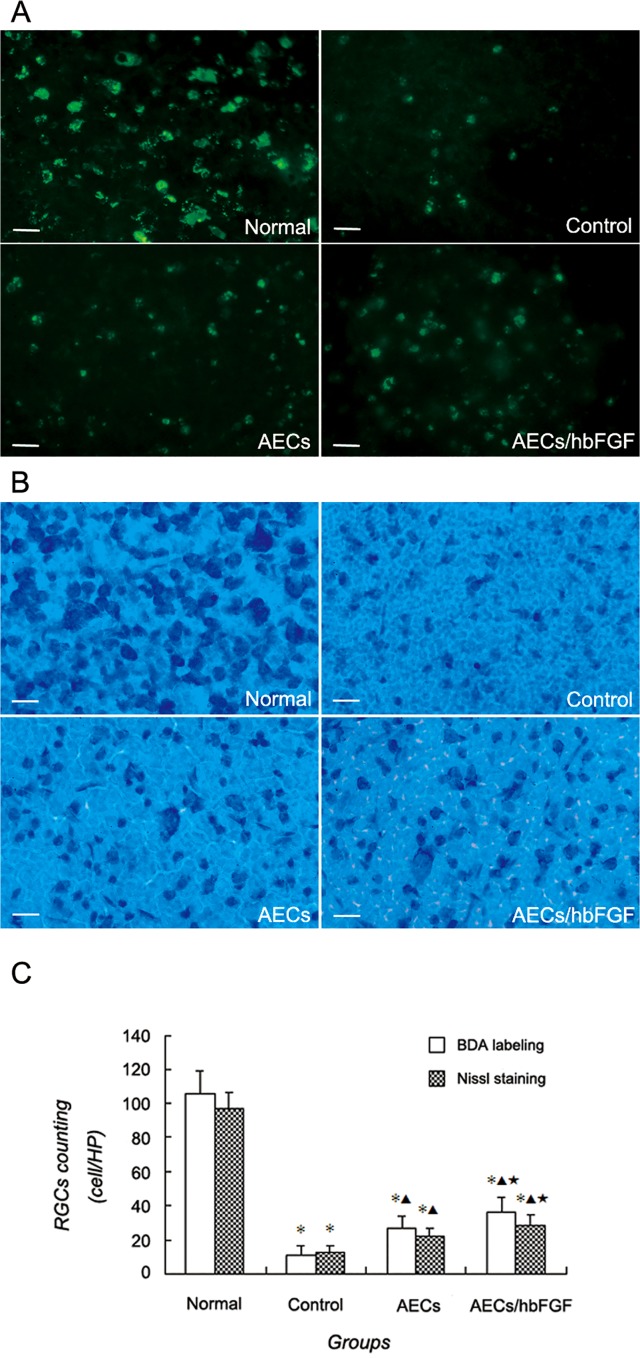
RGCs labeling and counting in retina flattening of different groups. A: RGCs labeled with BDA in different groups. Bar = 20μm. B: RGCs of Nissl staining in different groups. Bar = 20μm. C: Comparisons of RGCs counting in different groups (HP: High power field). (*: *P*<0.01 vs Normal group; ▲: *P*<0.01 vs Control group; ★: *P*<0.01 vs AECs group).

As shown in retina flattening after BDA labeling and Nissl staining respectively, 28d after surgery the RGCs in the normal group were homogeneously labeled with green fluorescein or toluidine blue, and the cell bodies were large and plump. Meanwhile the labeled RGCs in the control group and cell transplantation groups arranged disorderly, as well as large cell bodies could rarely be found (Fig. 4A,4B).

Variance analysis of RGCs counting revealed that the differences of RGCs counting of either BDA labeling or Nissl staining method respectively among the four groups were statistically significant (BDA labeling method: F = 529.51,*P*<0.0001; Nissl staining method: F = 468.42,*P*<0.0001). Furthermore, RGCs counting in the control group was lower than other groups (*P*<0.0001), and RGCs counting in the AECs/hbFGF group was higher than that of the AECs group (BDA labeling method: *P* = 0.002; Nissl staining method: *P*<0.0001) but was lower than that of the normal group (*P*<0.0001) (Fig. 4C).

### AECs Promoted GAP-43 Expression within Transected Optic Nerve

**Fig 5:**
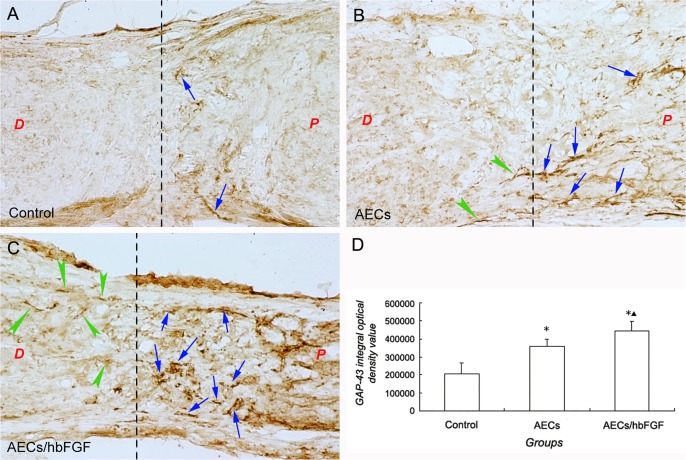
GAP-43 immunohistochemical staining in the injury site of optic nerve in different groups. A: GAP-43 staining in control group. B: GAP-43 staining in AECs group. C: GAP-43 staining in AECs/hbFGF group. (Arrowhead indicates GAP-43 immunoreactive substance; The dotted line indicates the junction of lesion zone and the distal part; “D” indicates “the distal part”, and “P” indicates “the proximal part”). D: Comparisons of the GAP-43 integral optical density value among different groups. (*: *P*<0.01 vs The control group; ▲: *P*<0.01 vs The AECs group).

The protein expression of GAP-43 was not visualized within the optic nerve of the normal group with immunohistochemical staining method 28d after surgery, while the expression of GAP-43 was visualized in other groups (Fig. 5A). In the control group, there was little expression of GAP-43 in the lesion zone of optic nerve, and none was found in the distal part next to the lesion zone. Meanwhile in the cell transplantation groups the GAP-43 immunoreactive substances were found only in the lesion zone but also in the distal part next to the lesion zone, which seemed to distribute as axon-like shape. Furthermore, the GAP-43 immunoreactive substances in the AECs/hbFGF group were visualized more obviously and even farther to the lesion zone than that of the AECs group in the distal part of the optic nerve.

By measuring the integral optical density value of the GAP-43 immunoreactive substance in the injured sites (including the lesion zone, the distal part and the proximal part), the result showed that the differences of the GAP-43 integral optical density value among the three groups were statistically significant (F = 20.34,*P*<0.0001). The GAP-43 integral optical density value in the control group was lower than the cell transplantation groups (*P*<0.0001), and the GAP-43 integral optical density value in the AECs/hbFGF group was higher than that of the AECs group (*P* = 0.0299) (Fig. 5B).

## Discussion

Optic nerve, also known as the second of twelve paired cranial nerves, transmits visual information from retina to brain. Since it is derived from the diencephalon during embryonic development, the optic nerve has generally been considered to be part of the CNS. Furthermore the optic nerve has often been used for investigating the CNS, especially for modeling axonal injury of the brain, due to its intrinsic structure and microenvironment differing from the peripheral nervous system as well as its easily accessible location [13]. The neurodegenerative process after optic nerve injury is much similar to the process of secondary degeneration in the CNS [14,15]. After optic nerve injury, part of the RGCs encounter early necrosis, furthermore a great number of the rest RGCs undergo delayed apoptosis [16,17]. However, even in a complete transection model, a few of RGCs are still able to survive for a long period of time after the injury [18,19]. Since the optic nerve is formed principally by axons of the RGCs, it is crucial to protect RGCs and reduce cell loss in order to restore neural function following optic nerve injury.

GAP-43 is a nervous tissue-specific cytoplasmic protein considered as a neuron specific marker of outgrowing axons, which is expressed at high levels in neuronal growth cones during development and axonal regeneration [20]. GAP-43 is characterized as the major growth cone substrate of protein kinase C (PKC), and it is indicated that GAP-43 plays an important role in neurite formation, regeneration, and plasticity. Therefore it could be considered as an intrinsic determinant of axon growth state [20–23]. Thus we chose GAP-43 to be a biochemical marker for growing axons, which could partly reflect the regeneration state of injured optic nerve.

Our study demonstrated that AECs/hbFGF could synthesize and secrete bFGF peptide, which could possibly promote AECs to survive and migrate after being transplanted into the injured optic nerve. The reason why we chose current method of damage to the rat optic nerve over the conventional cut with iridectomy scissors or crush with forceps was that it could provide a valid complete transection, while in this extremity no nerve fibers was remained within the gap. Moreover since the meningeal sheath was reserved well and vessels were kept intact, it was possible to inject cell suspension into the transverse gap and keep it. Therefore the effect of transplanted cells could be clearly observed and analyzed. Meanwhile, during the procedures there were displaced amacrine cells (dACs) being labeled by both Nissl staining and BDA staining, but we could possibly distinguish a majority of RGCs with dACs by morphological observation. Generally the cytoplasm of RGC is relatively big with obvious Nissl bodies after staining [5]. So we set counting criteria that only cells with an intact membrane profile and plump cytoplasm, as well as hyperchromatic Nissl bodies in Nissl staining or bright fluorescence within cytoplasm in BDA staining should be included. This counting procedure might exclude most of the dACs as well as a part of small RGCs without obviously stained Nissl bodies, however, with sticking to the same counting criteria in all groups, we could obtain a reliable result of differences through comparison among groups. According to the results, the AECs/hbFGF transplantation significantly reduced RGCs loss, as well as enhanced GAP-43 expression within the complete transected optic nerve. Moreover, comparing with AECs transplantation, AECs/hbFGF seemed to have played more positive role in protecting RGCs and promoting neural regeneration following optic nerve injury, indicating that bFGF gene modification enhanced the curative value of AECs as a candidate cell for cell therapy.

In view of the stem cell characteristics, secretion of several neurotrophic factors and low immunogenicity, as well as avoidance of ethical issues on the cell source, AECs has been considered as one of the promising cell types for transplantation strategies to treat neurological disorders. Okawa et al [10] proved that AECs could migrate, survive in ischemic brains of rat and differentiate like neuronal and neural stem cells. It was also reported that human AECs exhibit beneficial effects such as neurotrophism, rescuing axotomized neurons when transplanted into the injured spinal cord of bonnet monkeys, while immune reaction was not observed at the transplantation site after such xenogenic transplantation [11]. Though AECs produce several neurotrophic factors, such as epidermal growth factor [24], insulin-like growth factor [25], BDNF NT-3, there is no evidence that it is capable of synthesizing and releasing bFGF, which is a potent mitogenic factor for cells originated from mesoderm and ectoderm [26], and plays important roles in neurogenesis, axon growth, and differentiation [27]. Theoretically bFGF gene modification may enhance the trophic effects of AECs and make it more beneficial in neural regeneration as therapeutic cell. Meng et al. [28] reported that AECs modified with the bFGF gene could enhance neural stem cells survival in vivo and promote the survival of host neurons after being transplanted into rat model of spinal cord injury. Our study also supported the enhancement of neurotrophic function of AECs by bFGF gene transduction. It was shown that bFGF produced by AECs/hbFGF, combining with other factors that AECs synthesize, could promote axon regeneration at the lesion site of optic nerve that was completely transected with a clear gap—without any neural structure contiguous, resulting in a small part of the axons regenerate into even through the lesion zone. Meanwhile the bFGF and the co-factors produced by AECs/hbFGF may gather in retina by retrograde axoplasmic transport or migration of transplanted cells, promoting RGCs survival and reducing cell loss. Accordingly, the combination of AECs with bFGF may theoretically be beneficial in ameliorating the microenvironment of the injured optic nerve as well as protecting RGCs, enhancing neural regeneration.

In summary, bFGF gene modified rat AECs could promote RGCs survival and regeneration of axons in the completely injured optic nerve of rat, indicating their potential for cell therapy treating optic nerve injury and related diseases. However, the mechanisms underlying the regeneration process and visual function restoration afterwards needs further investigation.
