# Correction: Positive Effects of bFGF Modified Rat Amniotic Epithelial Cells Transplantation on Transected Rat Optic Nerve

**DOI:** 10.1371/journal.pone.0124713

**Published:** 2015-04-07

**Authors:** The PLOS ONE Staff

There are errors in the Funding section. The correct funding information is as follows: This work was supported by The National Basic Research Program of China (973 Program, Grant No. 2005CB724302) and National Natural Science Foundation of China (Grant No. 31271282). The funders had no role in study design, data collection and analysis, decision to publish, or preparation of the manuscript.
